# Confocal Laser Endomicroscopy in Resection of Sinonasal Malignant Melanoma—Preliminary Report on Real-Time Margin Assessment and Support in Surgical Decision-Making

**DOI:** 10.3390/jcm13154483

**Published:** 2024-07-31

**Authors:** Nina Wenda, Kai Fruth, Sebastian Wagner, Annette Fisseler-Eckhoff, Jan Gosepath

**Affiliations:** 1 Department of Otolaryngology, Head and Neck Surgery, Horst Schmidt Kliniken, 65199 Wiesbaden, Germany; kai.fruth@web.de (K.F.); jan.gosepath@helios-gesundheit.de (J.G.); 2 Department of Pathology, Helios HSK, 65199 Wiesbaden, Germany; sebastian.wagner@helios-gesundheit.de (S.W.); annette.fisseler-eckhoff@helios-gesundheit.de (A.F.-E.)

**Keywords:** sinonasal mucosal melanoma, confocal laser endomicroscopy, real-time imaging, optical biopsy, guided endoscopy

## Abstract

**Background/Objectives:** Building upon the rising value of Confocal Laser Endomicroscopy (CLE) in squamous cell carcinoma of the head and neck, we present the first application of CLE during the resection of sinonasal malignant melanomas. This study aims to evaluate the potential of CLE to assist surgeons in intraoperative decision-making, with a particular focus on resection margin assessment within the constrained nasal cavity. **Methods:** Two cases of sinonasal malignant melanoma were included in this study. CLE was employed to examine visible tumors and their margins, both pre- and post-endoscopic resection. The findings were compared to histopathological results as well as data on squamous cell carcinoma, for which malignancy criteria had already been established in prior projects. **Results:** CLE provided the real-time visualization of sinonasal malignant melanomas and their margins, successfully differentiating between healthy and neoplastic tissue compared to histopathological findings. **Conclusion:** CLE offers the potential for real-time assessment, aiding surgeons in more precise tumor resection and potentially improving patient outcomes. This study demonstrates the feasibility of using CLE in the resection of sinonasal malignant melanoma, highlighting its ability to differentiate between healthy and neoplastic tissue intraoperatively.

## 1. Introduction

Sinonasal mucosal melanoma (SNMM) represents a rare oncological entity that accounts for the majority of head and neck mucosal melanomas [1]. It typically presents with nonspecific symptoms such as epistaxis or nasal obstruction. The primary sites of origin are predominantly the nasal cavity, followed by the maxillary and ethmoid sinuses. In the great majority of cases, SNMM arises in the nasal septum or the lateral wall of the nasal cavity [2]. Patients diagnosed with SNMM often face poor survival outcomes with limited consensus on the optimal treatment strategies. While wide surgical excision is generally considered the primary treatment modality, the addition of postoperative radiation therapy is common to enhance locoregional control [3]. With the increasing availability of targeted therapies, the identification of genetic markers has become crucial, even though the mutation profile of SNMM remains incompletely characterized [4].

The rarity of SNMM has led to the scarcity of comprehensive literature detailing treatment approaches, leading to reliance on isolated case reports and retrospective series for guidance [5].

One of the most important prognostic factors for tumors of the nasal cavity and paranasal sinuses, including SNMM, represents the quality of tumor resection with negative surgical margins [6].

In recent years, Confocal Laser Endomicroscopy (CLE) has become an increasingly popular technology, advancing diagnostic capabilities in multiple medical fields. CLE provides real-time microscopic images of mucosal tissue. This capability allows surgeons to visualize tissue structures at a microscopic level during surgery, eliminating the need for traditional histopathological processing [7]. CLE has already found successful applications in gastroenterology [8], pneumology [9], urology [10], and neurosurgery [11], where it showed its potential to enhance diagnostic precision and guided therapeutic interventions. The dynamic assessment of tumor margins through CLE reduces the likelihood of leaving behind residual cancerous tissue during resection [12]. In prior projects, our group was able to validate the value of CLE in squamous cell carcinoma of the head and neck and establish malignancy criteria [13,14,15]. This experience has motivated further exploration of CLE’s potential in surgical treatment of SNMM.

The aim of this pilot study is to harness the potential of CLE to enable the real-time differentiation of healthy and neoplastic tissues in patients with SNMM. Specifically, we seek to evaluate the effectiveness of CLE in assessing surgical margins during SNMM resection. By providing immediate and accurate identification of tumor boundaries, CLE has the potential to enhance surgical decision-making, ensuring complete tumor removal while preserving as much healthy tissue as possible and therefore offering a promising perspective for improved patient care in the management of this rare malignancy.

## 2. Materials and Methods

This study aimed to investigate the application of CLE in the endoscopic resection of two cases of SNMM. Patients were enrolled from a tertiary medical center in Germany. This study was conducted following the principles outlined in the Declaration of Helsinki. Approval was obtained from the institutional review board (IRB). Written informed consent was obtained from both participants before their inclusion in the study.

The first patient was a 58-year-old female suffering from recurrent unilateral epistaxis. The second patient was an 84-year-old male with unilateral nasal obstruction and recurrent epistaxis.

**Figure 1:**
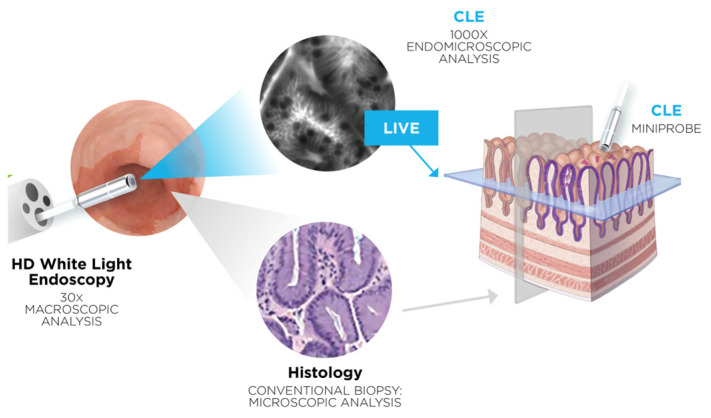
Overview of CLE functioning with kind permission for publication by Mauna Kea Technologies.

In CLE, a low-power laser targets a single point within a microscopic field of view. This system uses the same optical lens for both condensation and objective functions, effectively folding the optical path and aligning the laser illumination point precisely with the detection point within the specimen. This alignment of illumination and detection on the same focal plane defines the term “confocal”. The captured signals emitted from the illuminated point are translated into grayscale images of the tissue’s microscopic structure (see Figure 1).

The miniprobe units are highly adaptable, serving as independent tools that can be inserted through the working channels of various endoscope types. With the intravenous administration of 2.5 mL of fluorescein (100 mg/mL) as a contrast agent, confocal assessment becomes feasible in less than 30 s. In our study, we used a probe-based system (pCLE; Cellvizio^®^ Endomicroscopy System, Mauna Kea Technologies, Paris, France) featuring a 1.6 mm flexible miniprobe, offering a field of view with a diameter of 240 µm. This enables observations within a confocal depth range of 55–65 µm with a resolution of 1 µm and a 1000-fold magnification.

**Figure 2:**
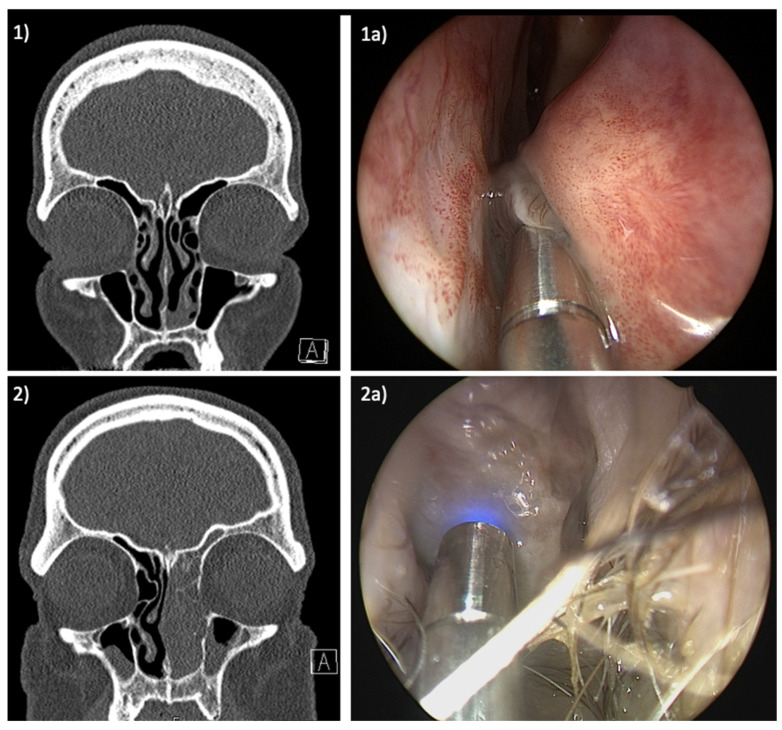
Radiologic and endoscopic findings: (**1**) CT-scan of patient I with SNMM of the floor of the left nasal cavity and the lower part of the inferior turbinate, (**1a**) endoscopic view of the laser probe applied on the tumor, (**2**) CT-scan of patient II with SNMM completely obstructing the left nasal cavity, (**2a**) endoscopic view of the laser probe applied on the tumor in the left vestibule.

The study cohort comprised two patients diagnosed with SNMM of the left nasal cavity and scheduled for surgical resection. Figure 2 displays the radiological and endoscopic findings of both patients. CLE was initially employed prior to resection to scrutinize the margins of the lesions, with the aim of visualizing the extent of the tumor. Subsequently, after the resection, CLE was once again utilized to assess the surgical site, focusing on the presence of any residual tumorous cells in the area of the resection margin.

During the endoscopic resection, approximately 5 min of CLE video was captured for each patient, from which several hundred images were extracted. Due to the presence of artifacts and variations in image quality, we selected 20 high-quality images per patient for further evaluation. These images were categorized into four groups: tumor center, macroscopic tumor margin, healthy mucosa, and resection margins, with five images in each category. These images were then presented to two separate pathologists for comparison with corresponding biopsy samples obtained from the same areas. The pathologists assessed the images for the presence of tumor cells and their agreement with histopathological findings. This evaluation involved the identification of cellular characteristics, an assessment of CLE image quality, and a level of agreement with conventional histopathological hematoxylin and eosin (H&E) staining.

Following the resection, both patients reported complete regression of symptoms and have been on regular follow-up for over one year at present.

## 3. Results

The implementation of CLE proved to be consistently feasible across the endoscopic resection of both tumors, with initiation approximately 30 s post-intravenous administration of fluorescein. No unexpected side effects resulting from the administration of the contrast agent could be observed. The CLE examination added approximately 10 min to the respective procedure.

Although the image quality varied depending on factors like tumor location, tissue vascularization, and tissue vulnerability, high-quality images could be captured in all examined areas. The positioning of the laser probe was easily feasible when examining the nasal septum, the floor of the nasal cavity, and the tumors themselves. Notably, with the assistance of rigid instruments, successful probe insertion within constrained regions such as the lateral nasal wall beneath the remnants of the inferior turbinate was achievable.

**Figure 3:**
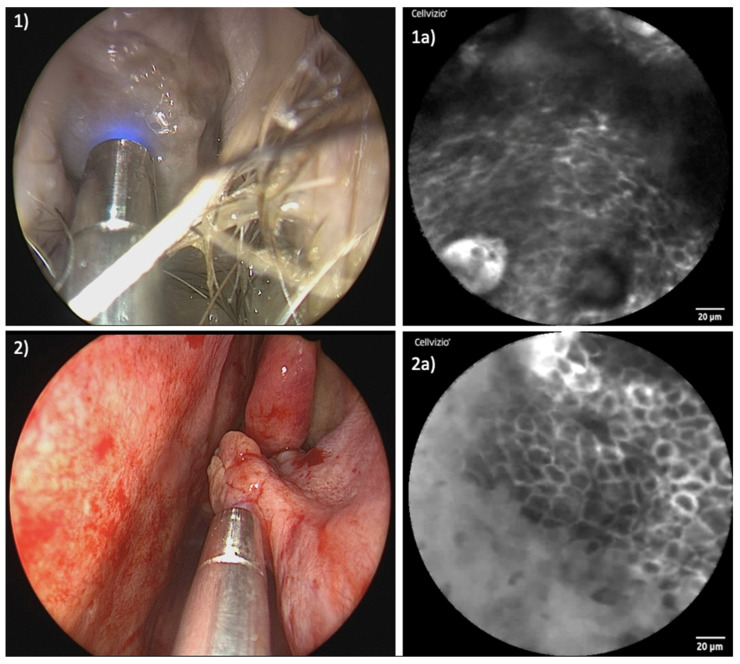
Examination of patient I: (**1**) Endoscopic view of examination of tumor center; (**1a**) corresponding CLE image with blurred, mainly extinguished cell borders, formation of vascular clusters, vasodilation, and inhomogeneous distribution of fluorescein; (**2**) laser probe examining healthy tissue of inferior turbinate; (**2a**) corresponding CLE image displaying regular cellular architecture, clear cell margins, and homogenous uptake of fluorescein.

**Figure 4:**
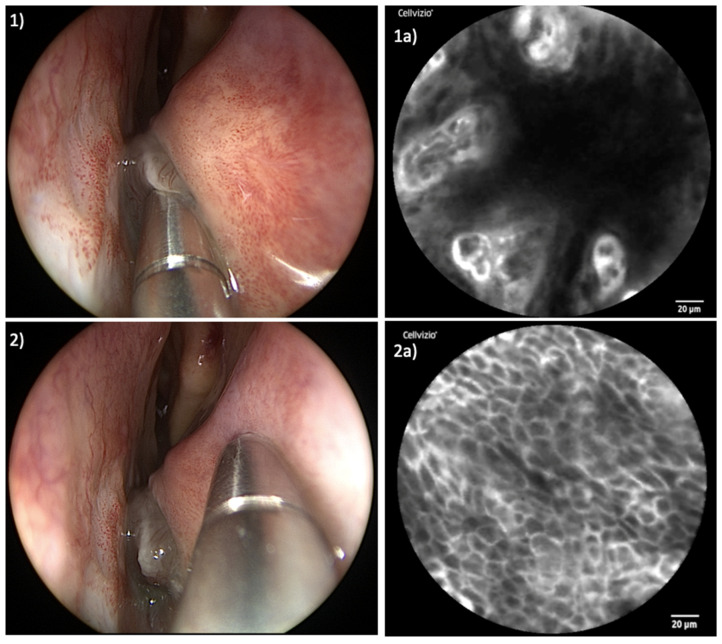
Examination of patient II: (**1**) Endoscopic view of examination of tumor center; (**1a**) corresponding CLE image presenting enlarged capillary, irregular cellular architecture, and distinctly reduced fluorescein uptake; (**2**) laser probe examining healthy tissue of remnants of left inferior turbinate; (**2a**) corresponding CLE image presenting neatly organized cells with clear cellular margins and homogenous uptake of contrast agent.

Figure 3 and Figure 4 show a comparison of the endoscopic and confocal findings in the area of the tumor center and the healthy marginal area in both patients.

Exploring healthy mucosal zones surrounding the tumors, consistent patterns could be observed. The cellular configuration presented uniformly, accompanied by well-defined cell margins and a homogenous uptake of fluorescein.

As expected, a transformation occurred in the area of the tumors. The cellular architecture exhibited disarray, deviating from the organized structure observed in healthy tissue. Simultaneously, capillaries within tumor sites showed qualitative and quantitative increases. The uptake of fluorescein within tumor areas appeared uneven, resulting in a diverse array of irregular cellular patterns. Supplementary Video S1 shows parts of the real-time examination of patient I, starting in the healthy part of the mucosa of the inferior turbinate. From second 21 you can see the transition from healthy to malignant tissue with a line-up of dilated capillaries. The video sequence has been slowed down for better understanding.

The findings from CLE were verified via histopathological assessments. In total, 40 CLE images were evaluated by the pathology experts. For the 10 images depicting healthy mucosa, the experts correctly identified all 10 images. Of the 10 images from the tumor center, 8 were correctly identified as containing tumor cells, while 2 images were marked as uncertain; none of these images were misclassified as healthy mucosa. The images from the macroscopic tumor margin were more challenging, with 7 out of 10 correctly identified as containing tumor cells and 3 deemed uncertain, but again, none were classified as healthy mucosa. The evaluation of the resection margins proved to be the most difficult due to artifacts such as blood and coagulation remnants. Here, 6 out of 10 images were correctly identified as healthy mucosa, 3 were marked as uncertain, and 1 image was misclassified as containing tumor cells, despite histopathological evaluation showing no residual tumor cells.

**Figure 5:**
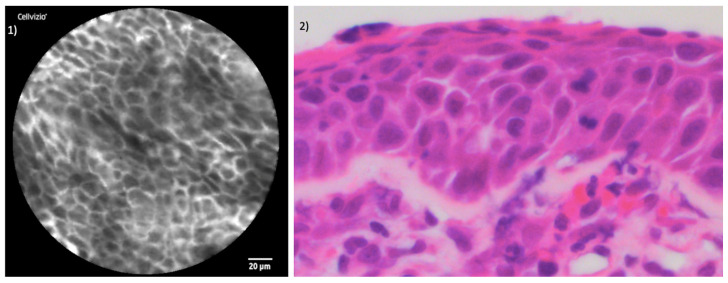
Comparison of CLE and histopathological sections in healthy tissue: (**1**) CLE image of healthy endonasal epithelium; (**2**) histopathological cross-section of regular endonasal squamous epithelium hematoxylin and eosin (H&E) staining.

**Figure 6:**
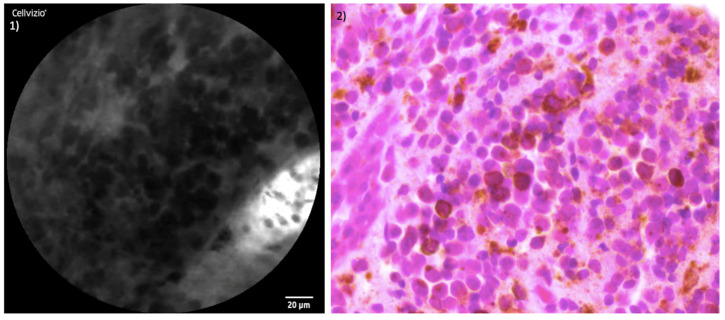
Comparison of CLE and histopathological sections SNMM of patient I: (**1**) CLE image of SNMM; (**2**) corresponding histopathological cross-section H&E staining.

**Figure 7:**
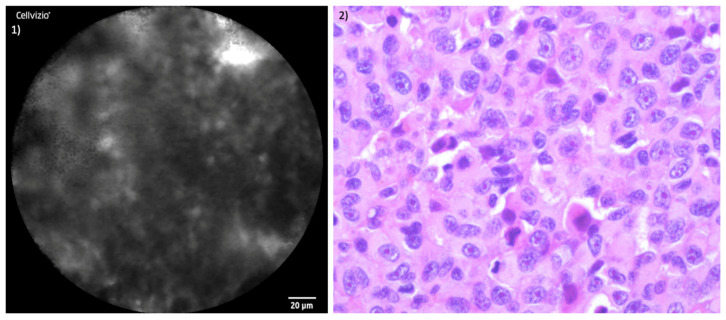
Comparison of CLE and histopathological sections SNMM of patient II: (**1**) CLE-image of SNMM; (**2**) corresponding histopathological cross-section H&E staining.

The juxtaposition of CLE images and the histopathological section of healthy mucosa is presented in Figure 5. Figure 6 and Figure 7 illustrate the side-by-side comparison of CLE images and their corresponding histopathological sections of both tumors.

## 4. Discussion

Despite advances in therapeutic strategies, SNMM continues to present a poor prognosis. In contrast to the 5-year overall survival rate of 70–80% for localized cutaneous melanoma, patients with localized SNMM face a markedly lower 5-year overall survival rate, ranging from 20% to 55% [1]. This poor survival rate is attributed mainly to distant metastatic disease [16].

The lack of a clear consensus on the optimal management of SNMM further complicates the clinical landscape. Although wide surgical excision is generally considered the primary treatment modality, its efficacy is limited, and the need for additional strategies is evident. Postoperative adjuvant radiation therapy is frequently employed to enhance locoregional control [17].

Furthermore, there is ongoing discussion about surgical strategies in SNMM management. Endoscopic approaches demonstrate advantages in terms of a shorter duration of surgery and fewer complications but also present superior post-operative cosmetic effects with minimal scarring compared to open approaches. Moreover, there are no discernible differences in local recurrence, disease-free survival, or overall survival rates when comparing the surgical approaches [18].

To our knowledge, this is the first study evaluating the benefits of CLE in diagnostic and therapeutic strategies of SNMM. This study demonstrates the feasibility of this technology during endoscopic resection, providing real-time insights into tumor margins and cellular characteristics. The capacity of CLE to differentiate healthy and neoplastic tissue, as well as its ability to aid in surgical decision-making, positions it as a promising tool in the surgical management of SNMM.

CLE relies on fluorescence contrast agents such as intravenously administered fluorescein sodium (2.5 mL of 100 mg/mL fluorescein 10%). It permeates the mucosa, creating strong contrast within the connective tissue and capillary network by binding with serum albumin. Unbound fluorescein molecules traverse capillaries, illuminating the extracellular matrix and enabling CLE examinations within seconds post-injection [19]. It is widely used in ophthalmology with a well-established safety record [20].

CLE presents several technical challenges and user-related limitations. Mastering the manipulation of the laser probe requires a learning curve, emphasizing the importance of surgeon familiarity with the technique. Achieving optimal image quality depends on correct contact pressure and angle, varying across different anatomical regions. Imaging artifacts due to blood and remnants of coagulation were particularly demanding in the examination of the resection margins, representing the most critical part of the examination, as the goal is to ensure no residual tumor remains.

Even for pathologists, the interpretation of CLE images and verification of comparability to histopathological sections is a challenging task. Most importantly, the grayscale images represent a horizontal plane of the mucosa, perpendicular to typical histopathological sections.

Our pathologists demonstrated high accuracy in identifying healthy mucosa and a good ability to detect tumor cells, particularly in images from the tumor center and macroscopic tumor margin. However, the evaluation of resection margins remains challenging due to the presence of artifacts.

The evaluation of CLE sensitivity and specificity in the head and neck region varies among research groups.

Using a topically applied contrast agent instead of systemically applied fluorescein, Abacci et al. reported sensitivity and specificity of CLE imaging at 73.2–75% and 30–57.4%, respectively [21]. This raises the question of the extent to which the results are comparable, particularly with regard to the achievable image quality.

A meta-analysis of six studies involving 213 patients with oral squamous cell carcinoma (OSCC) examined 361 lesions with CLE, demonstrating strong sensitivity and specificity at 95% and 93% and thus supporting the diagnostic value of CLE in OSCC [22].

Frenken et al. recently investigated the endonasal use of CLE including six patients with both inflammatory and neoplastic diseases. Their findings demonstrated a promising overall accuracy of 84.1%, sensitivity of 85.4%, specificity of 83.1%, positive predictive value of 72.5%, and negative predictive value of 92.1%. With a Fleiss’ κ value of 0.62, they additionally achieved a substantial level of agreement among the raters [23].

A scoring system for CLE evaluation implemented by Sievert at al. analyzed factors like tissue homogeneity, cell size, borders and clusters, capillary loops, and the nucleus/cytoplasm ratio in pharyngeal and laryngeal neoplasms [24]. Importantly, their study differentiated between CLE experts and non-experts, presenting distinct outcomes. CLE experts achieved impressive accuracy, sensitivity, and specificity of 90.8%, 95.1%, and 86.4%, respectively. Non-experts, while slightly lower, still demonstrated respectable scores of 86.2%, 86.4%, and 86.1%. The different results between CLE experts and non-experts underline the importance of familiarity with the technology. For optimal results, both the surgeon handling the probe and the pathologist interpreting the images must be well-versed in CLE. Surgeons will need to learn how to reliably capture high-quality images of the examined region, supported by pathologists accurately interpreting and analyzing these images. One future focus should therefore rely on training teams of surgeons and pathologists to interpret the CLE images, thus supporting surgical decision-making, particularly when it comes to the further resection of macroscopically clear margins.

In 2022, Sievert et al. successfully transferred their scoring system from pharyngeal and laryngeal tumors to tumors of the oral cavity [25]. While this transfer of findings to another anatomical region of the head and neck is promising, it is important to note the differences in the variety of tumor entities. In the larynx, pharynx, and oral cavity, squamous cell carcinoma (SCC) is predominant. In contrast, tumors of the nasal cavity, the paranasal sinuses, and skull base present a significantly greater variety of entities, including SNMM.

So far, the majority of studies investigating the use of CLE in the head and neck region have almost exclusively focused on the examination of SCC. The first use of this technology in SNMM revealed a remarkable congruence in distinguishing between healthy mucosal tissue and neoplastic areas. The established malignancy criteria validated in SCC, including an irregular cellular architecture, the heterogeneous distribution of the contrast agent, and vascular alterations, demonstrated satisfying applicability in SNMM, enabling the successful identification of tumor margins during endoscopic resection.

Although challenging due to the considerably lower incidence of these tumors, future studies need to delineate specific criteria that differentiate between SCC and SNMM alongside other endonasal neoplasms using CLE. This pursuit holds the potential to enhance the precision of CLE, not only in discerning between benign and malignant tissues but also in distinguishing among different malignancies.

Obviously, the small sample size of only two patients does not provide sufficient data for robust statistical analysis. Nevertheless, the number of images generated per patient appears adequate for a preliminary proof of concept. Future studies involving larger cohorts are necessary to validate these findings and to refine the technique for clinical application. The promising results from this preliminary report encourage continued investigation into the use of CLE to improve surgical outcomes and diagnostic accuracy in sinonasal malignant melanoma.

## 5. Conclusions

This pilot study highlights the potential utility of CLE in the surgical treatment of SNMM, demonstrating its capability for real-time mucosal imaging and its potential to aid in surgical decision-making. While the initial findings are promising, it is important to acknowledge that the evidence is still limited. Therefore, conclusions regarding the clinical value of CLE should be considered preliminary. Further studies with larger cohorts are necessary to verify these results and fully establish the role of CLE in improving diagnostic precision and surgical efficacy in SNMM.

## Data Availability

The original contributions presented in the study are included in the article/Supplementary Material, further inquiries can be directed to the corresponding author/s.

## Supplementary Materials

The following supporting information can be downloaded at https://www.mdpi.com/article/10.3390/jcm13154483/s1: Video S1: Real-time examination of patient I using CLE.
